# Acute nutritional stress during pregnancy affects placental efficiency, fetal growth and adult glucose homeostasis

**DOI:** 10.18632/oncotarget.22695

**Published:** 2017-11-25

**Authors:** Sajida Malik, Alan Diot, Karl Morten, Eszter Dombi, Manu Vatish, C.A. Richard Boyd, Joanna Poulton

**Affiliations:** ^1^ Nuffield Department of Obstetrics and Gynaecology, University of Oxford, Oxford, UK; ^2^ Department of Physiology, Anatomy and Genetics, University of Oxford, Oxford, UK

**Keywords:** placenta, fetal weight, stress, placental efficiency, glucose homeostasis

## Abstract

Exposure to maternal malnutrition impairs postnatal health. Acute nutritional stress is less clearly implicated in intrauterine programming.

We studied the effects of stressing pregnant mothers on perinatal growth and adult glucose homeostasis. We compared one group (“stressed”, mothers fasted for 16 hours) with controls (“unstressed”). We found that fasting stress had adverse effects on the weight of the fetuses conceived (*p*<0.005) and the placental efficiency (*p*<0.001) in stressed compared to unstressed offspring. Placental weight was increased (*p*<0.001) presumably in compensation.

Stress affected the glucose homeostasis of the offspring when they became adults (*p*<0.005) when analysed as individuals.

We previously linked nutritional stress throughout pregnancy with a mitochondrial stress response. We modelled placenta with cultured human trophoblast cells (BeWos) and fetal tissues with mouse embryonic fibroblasts (MEFs). High throughput imaging showed that the mitochondria of both cell types underwent a similar sequence of changes in morphology, induced by nutritional stresses.

The contrasting stress responses on fetal and placental weight were not captured by the cellular models. The stress of maternal fasting may be an important determinant of perinatal outcome in the mouse and might be relevant to nutritional stress in human pregnancy.

## INTRODUCTION

Fetal growth is a major determinant of perinatal mortality and postnatal health [1]. The Dutch famine [2] and subsequent studies [3, 4] have shown that aspects of intrauterine nutrition may reduce placental “efficiency” (the ratio of birth weight to placental weight) [5] and cause adverse phenotypes in postnatal life, such as metabolic syndrome. The effect of other types of stress on human offspring has not been easy to assess because it is frequently confounded by other factors such as maternal diet, smoking and social deprivation. Second-trimester maternal psychological stress increases the risk of infants being born small for gestational age [6]. In rodent studies, the effects of repeated stress on fetal growth may similarly be confounded with the effects of the maternal weight loss that it causes. Nevertheless, animal experiments demonstrate that *in utero* stress can permanently re-program the hypothalamic-pituitary axis (HPA), and this can have significant post-natal neuro-behavioural sequelae [7].

Effective fetal growth requires adequate maternal nutrition coupled to active transport of nutrients across the placenta, which, in turn requires ATP much of which is generated by mitochondria. We previously developed a mouse model to explore the links between mitochondrial function and maternal protein intake in programming fetal growth and post-natal glucose homeostasis [8]. In order to emulate maternally inherited mitochondrial dysfunction affecting both mother and offspring, we used the anti-AIDS drug, zidovudine (AZT) that inhibits mitochondrial DNA (mtDNA) replication and hence causes mtDNA depletion [8]. We also administered a low protein diet (LPD), which has been linked to insulin resistance in later life. We compared pregnant dams exposed to one of three treatments: 1) LPD 2) AZT on a normal protein diet (so NPDAZT) 3) AZT and LPD (LPDAZT) with each other and with controls (NPD). All three treatments reduced neonatal weight. Further, AZT increased postnatal fasting glucose and the mean beta-cell area/islet was reduced in the LPD + AZT group compared with controls. Our major conclusion was that mitochondrial dysfunction exacerbates the effect of LPD on reduced neonatal weight, impairing islet development and postnatal glucose homeostasis [8].

In a later publication we linked maternal low protein diet throughout pregnancy with a stress response in placental mitochondria [9]. The low protein diet was sufficient to reduce individual fetal fresh weights and placental dry weight. The least successful litters had low placental efficiency but raised placental ATP and mtDNA content. We suggested that these changes implicated a stress response known as stress-induced mitochondrial hyperfusion (SIMH) and involves mitochondrial elongation [10]. It is associated with reduced mitophagy (recycling of damaged mitochondrial fragments) [11] and hyperpolarisation of mitochondria. It could thus underline the increased cellular ATP that we documented in placenta of mothers on a LPD [10]. Further we previously postulated that while this SIMH may confer an epigenetic benefit acutely, it is likely to disadvantage mitochondria by impairing the quality of mtDNA in the longer term. This likely accounts for the additive effects of LPD (causing SIMH) and AZT (causing mtDNA depletion and potentially increased point mutations) on neonatal weight (reduced) and glucose homeostasis (increased fasting glucose) [8].

Knowing that fibroblast cultures respond to 8-24 hours culture in glucose-free media with this SIMH response, we sought to model this *in vivo*. A response such as SIMH might be able to compensate for short-term maternal nutrient deprivation in mid pregnancy but might exacerbate the adverse effects in our model. We therefore fasted mothers for 16 hours between days E12 and E13. We anticipated that SIMH would already be present in the offspring of mothers already on LPD and their compensatory response to the stress of maternal fasting might thus be affected. We assessed the effect of maternal diet and mitochondrial (dys) function on fetal and placental weights in mice at embryonic day E18 and on live offspring from parallel litters on fasting glucose aged 20 weeks. All treatments were continued throughout postnatal life, as previously [8]. We report a major effect of the stress of maternal fasting (henceforth “stress”) on fetal weights and placental efficiency as well as glucose homeostasis in the adult (20 week) offspring, comparing stressed litters to the same experiment carried out over the preceding months in which mothers were not fasted (unstressed). Stressed mothers had dramatically reduced birth rate and fetal weights, compared to controls. Furthermore, placental efficiency and glucose homeostasis in the offspring were impaired.

We previously used trophoblast-derived cultured cells to model the effects of LPD on placenta [9]. We found that amino acid deprivation lengthened trophoblast mitochondria, consistent with a mitochondrial stress response that may underlie the low dry weight, raised ATP and impaired function of placenta in this mouse model. In that study, we documented changes in placental weight and water content that were reflected by, but less extreme, in fetuses. This contrasts with the present study where the short term nutritional stress changed fetal and placental weight in opposite directions. Hence we considered the possibility that mitochondrial stress responses might differ in fetal and placental tissues. We therefore went on to use cell culture models to compare mitochondrial stress responses in cell types representative of placenta and fetus to explore potential differences between them.

## RESULTS

### Pregnancy rate and litter size

Mothers were fasted overnight once during pregnancy on day E12-13. Overall 9/44 (20.4%) litters reached term, which is similar to the rate for unfasted (unstressed) mothers (41/177 (23%)). Stress had no significant effect on litter size.

**Figure 1:**
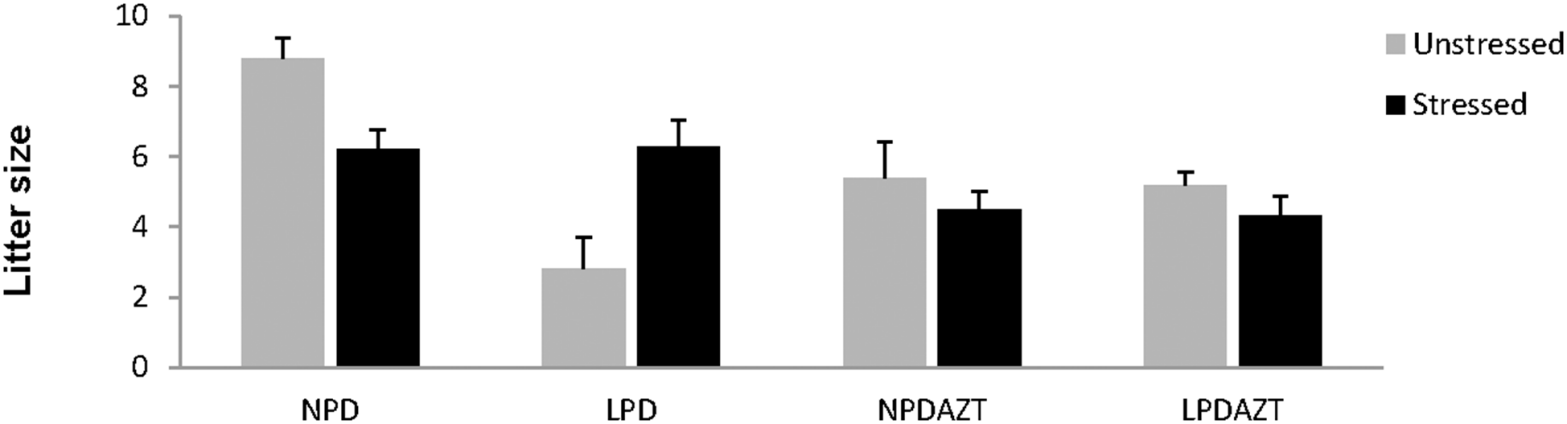
Litter size at E18 and maternal fasting Stress did not have a simple effect on litter size. It interacted significantly with the effects of LPD and AZT on litter size. Error bars are 1SE. Data is from 5, 5, 5 and 5 unstressed and 12, 9, 12 and 14 stressed litters corresponding to NPD controls, LPD fed on low protein diet, NPDAZT treated with AZT but on normal diet, LPDAZT fed on low protein diet and treated with AZT respectively.

The average litter size was however reduced by AZT (p=0.02, Figure 1).

### Fetal and placental weights

**Figure 2:**
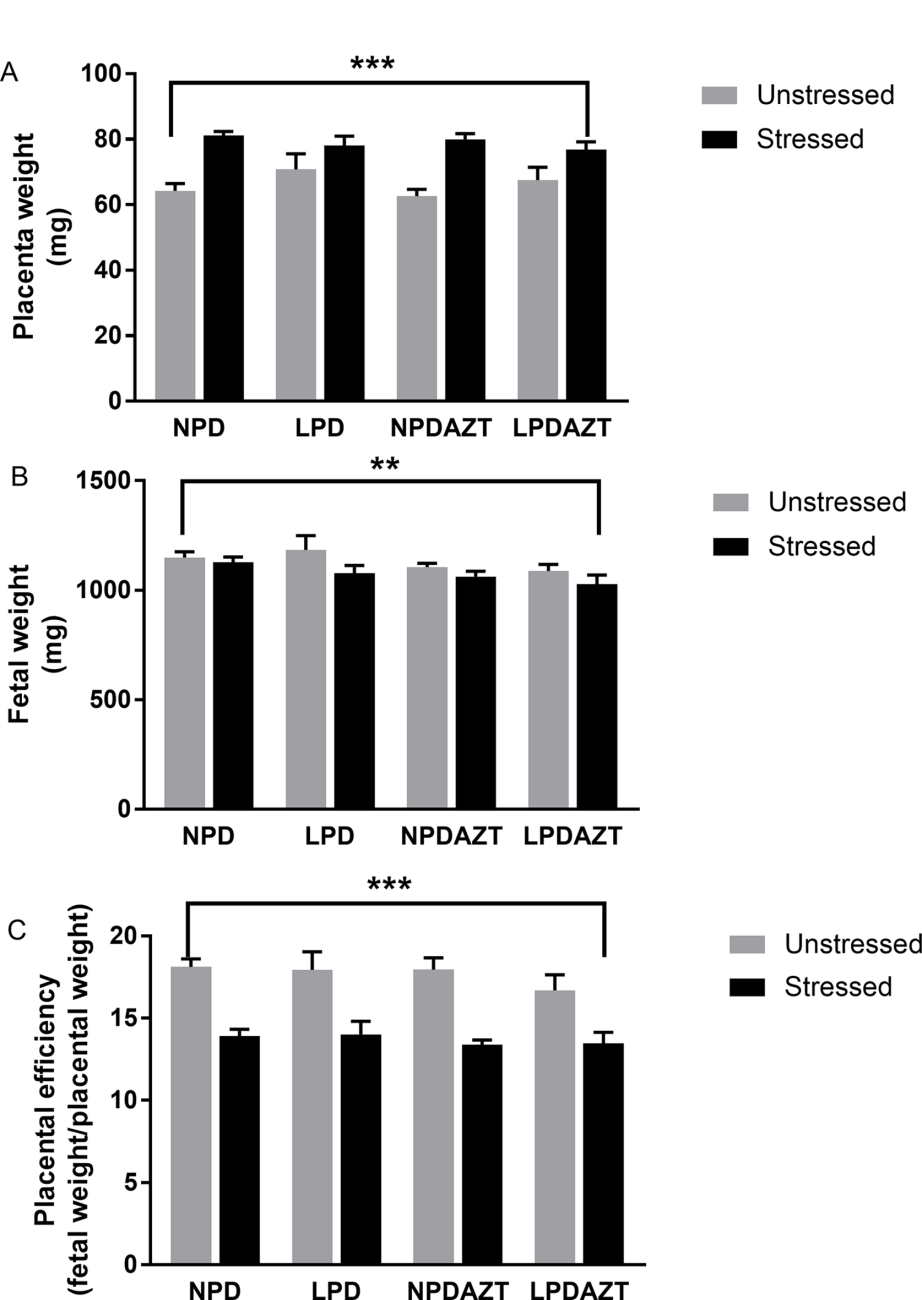
Placental weight increased and fetal weight and placental efficiency are reduced at E18 by maternal fasting **(A)** ANOVA showed that placental weight is increased by maternal stress (*p*<0.001) in all 4 conditions. **(B)** Fetal weight is reduced by maternal stress (*p*=0.005) in all 4 conditions (averaged across the average for each litter). **(C)** The ratio of fetal to placenta weight (a surrogate for the efficiency of placental transport) was reduced by maternal stress (*p*<0.001). This ratio partly adjusts for the interacting effects of litter size in such studies. N=44, 14, 27 and 26 in unstressed and 34, 31, 31 and 35 in stressed offspring from 5, 5, 5 and 5 unstressed and 12, 9, 12 and 14 stressed litters corresponding to control, LPD, NPDAZT and LPDAZT respectively. NPD controls, LPD fed on low protein diet, NPDAZT treated with AZT but on normal diet, LPDAZT fed on low protein diet and treated with AZT. Error bars are 1SE. Calipers represent ANOVA F test *p*-value for effect of maternal stress, ^***^p<0.001, ^**^p<0.01.

Figure 2 shows that maternal fasting increased placental weight (Figure 2A *p*<0.001) and reduced fetal weight (Figure 2B, *p*<0.005) in the offspring of the fasted mothers (averaged over litters, so that the dam is the unit of experimentation) in all 4 conditions (see Supplementary Figures 1 and 2 for raw data). Hence we then considered placental efficiency (which we define as fetal weight divided by placental weight). In our previous study we found that summed placental and fetal weights were greatest in large litters in which individual placental and fetal weights were reduced. We found that using the ratio of placental to fetal weight partly adjusts for the effect of litter size. Figure 2C shows that placental efficiency was significantly reduced by maternal stress (*p*<0.001, see Supplementary Figure 3 for raw data). These effects were not explained by reduced litter size, nor by the effects of LPD and AZT.

### Fasting glucose in the offspring

**Figure 3:**
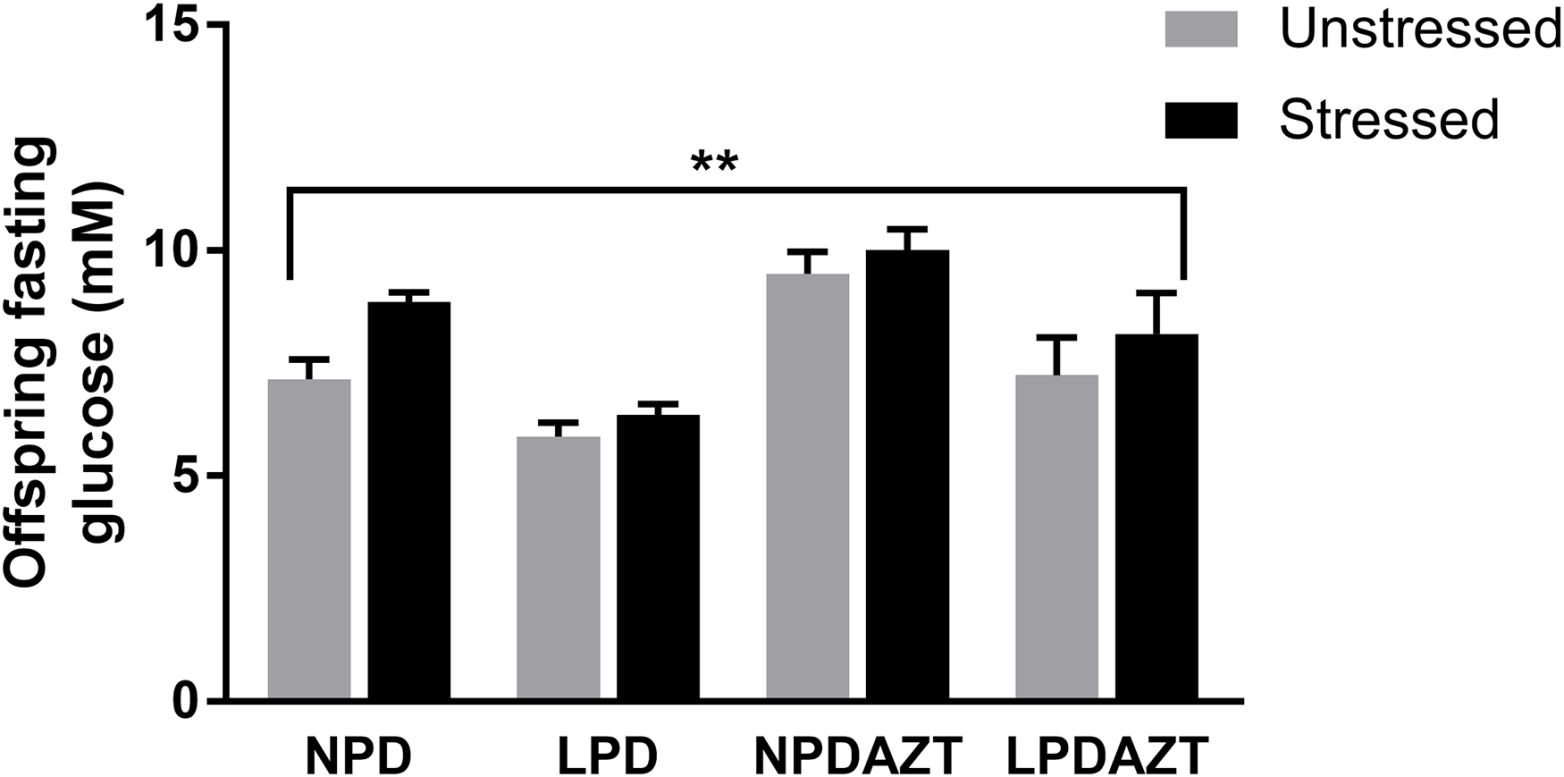
The fasting glucose of the offspring was significantly increased by maternal stress (p<0. 002) N=36, 21, 32 and 6 in unstressed and 16, 9, 10 and 15 in stressed offspring from 5, 4, 5 and 2 and 4, 2, 3 and 5 litters corresponding to control, LPD, NPDAZT and LPDAZT respectively. NPD controls, LPD fed on low protein diet, NPDAZT treated with AZT but on normal diet, LPDAZT fed on low protein diet and treated with AZT ANOVA showed that fasting glucose measurements in the offspring of the stressed mothers were significantly higher than those who were not fasted (*p*=0.002) and gender was also significant (*p*<0.01). Error bars are 1SE, ^**^p<0.01.

Postnatal adult glucose homeostasis, determined by fasting glucose was affected (Figure 3 *p*=0.002. ANOVA) in stressed compared to unstressed offspring, using individual mice as units (see Supplementary Figure 4 for raw data). LPD, significantly reduced and AZT increased postnatal fasting glucose, as previously *p*<0.05 and 0.001 respectively. As expected, the effect of gender was also significant (*p*<0.01, not shown), fasting glucose being higher in males. When sexes were analysed separately, the effect of stress on fasting glucose remained significant in females but not males (*p*=0.008 and NS respectively by ANOVA). When the analysis was repeated with litters as the unit of experimentation LPD and AZT remained significant, but stress and sex did not.

### Mitochondrial response to nutritional stress in tissue culture models

**Figure 4:**
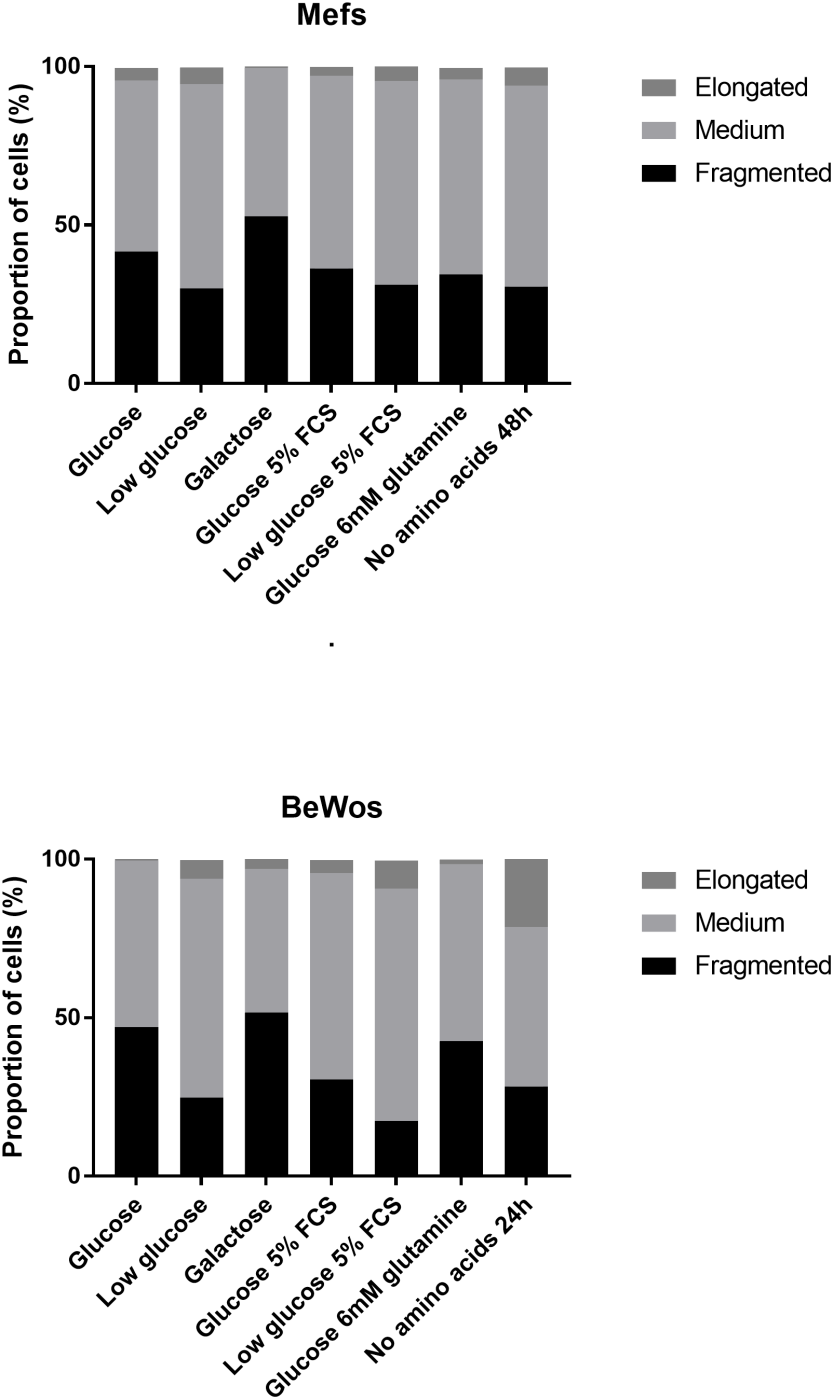
Similarities in the mitochondrial response of cultured cells derived from trophoblast and embryo to nutritional stresses The figure shows stacked bar chart illustrating the distribution of mitochondrial length for 7 different media (media deficient in glucose, amino acids or fetal calf serum) in MEFs (top) and BeWo cells (bottom). In the case of amino acid free media, exposure was for 24 and 48 hours duration for BeWos and MEFs respectively, as longer exposures were poorly tolerated. All other exposures were 3 days. For each condition >1100 cells were imaged and the mean mitochondrial length for each calculated. For means of each of these distributions see Supplementary Table 1.

Having previously linked mitochondrial stress responses with effects on placental weight and efficiency we sought to investigate this in cultured cells, representative of placenta and fetus. We modelled placenta with cultured human trophoblast cells (BeWos) and fetal tissues with MEFs in order to explore potential differences between these tissues. Both cell types were cultured in a variety of media (media deficient in glucose, amino acids or fetal calf serum for up to 3 days) and mitochondrial morphology assessed by high throughput imaging [12] (Figure 4). Neither cell type thrived in media in which amino acids had been removed, and BeWos were the more sensitive.

As previously reported [9], amino acid starvation elongated mitochondria as did low glucose, low fetal calf serum (5%) and media enriched in glutamine. Where glucose was removed from the medium and galactose used as the carbon source, mitochondria appeared fragmented compared with regular 25mM glucose. More detailed analysis showed that fragmentation at 3 days was preceded by elongation at 24-48h. Inspection of Figure 4 and the Supplementary Table 1 shows that though the starting morphology was different (BeWo mitochondria being longer) the direction of change in mitochondrial morphology was similar for both cell types. Each experiment was carried out in triplicate and a representative run is shown in Figure 4.

We were unable to detect differences in the mitochondrial response to nutritional stress over 0-3 days that might reflect the differences documented in response of placental and fetal weights to stress.

## DISCUSSION

We found that the stress engendered by a 16 hour fast on E12-13 during pregnancy adversely affected fetal weight and placental efficiency, that is, ratio of fetal to placental weight (*p*<0.005 and *p*<0.001 respectively, Figure 2). Placental weight, which is known to increase in compensation for hypoxia [13], was increased (*p*<0.001). It also increased fasting glucose in the offspring (*p*=0.002, Figure 3). These effects were not explained by reduced litter size, nor by the significant effects of LPD and AZT. While the effects of nutritional stresses on trophoblast and fetal mitochondria in tissue culture model were grossly similar (Figure 4), the effects of maternal fasting stress on placental and fetal weights were in opposite directions.

To our knowledge, this is the first study linking stress in pregnancy with all three parameters that are central to fetal programming: fetal weight, placental efficiency and adult glucose homeostasis [14]. Fielder et al [15] showed that fasting for 24 and 48 hours resulted in a significant increase in mouse placental lactogen-II on day 15 of pregnancy, and that fetal weights were significantly reduced after a 48-hour maternal fast. Animal experiments show that both repeated stress and impaired maternal nutrition are inter-related factors that can permanently affect the fetus. Stress affects maternal glucocorticoid metabolism [7] and blood pressure [16], and this in turn affects the weight and health of the offspring [17]. In our model of mitochondrial intra-uterine programming, maternal stress, caused by fasting, reduced placental efficiency. While it is clear that glucocorticoids play a key role [18, 19], there has been scanty direct evidence that a single stressful episode in pregnancy can impair placental efficiency.

Previous studies have shown that mild stresses, such as exposure to strobe light for 2 hours in mid pregnancy, can re-programme the HPA [20]. Nutritional stress may affect placental physiology [21] through this system, and this is potentially important both in animal models [16] and human disease [17]. Nevertheless maternal fasting for 16 hours on day E12-13 exerted pronounced effects on the offspring. In particular, rodents appear to have an increased appetite, doubling their food intake during pregnancy [22]. Further studies are needed to determine the critical period for the effect that we observed. Other investigators have implicated the HPA in the response to stress both in mid- gestation [23] and later, when the fetal HPA begins to release its own adrenocorticotropic hormone (ACTH) and corticosterone [24]. In addition to mechanisms potentially involving glucocorticoids, we speculate that perfusion could be affected, as major changes in murine placental vasculature and fetal nutrition occur between day E10.5 and 12.5 [25] (corresponding to around week 11 in humans [26]).

In our previous studies we found that mitochondrial dysfunction appeared to exacerbate some of the effects of low protein diet, though not the relationship with postnatal fasting glucose. In this previous work we found that LPD decreased litter size and neonatal weight [8]. Furthermore, early exposure to AZT interacted with LPD to impair fetal development and post-natal glucose homeostasis [8]. Glucose homeostasis was impaired in both the NPDAZT, and LPDAZT groups, and beta-cell area/islet was reduced [8]. We also implicated SIMH in some of the effects of LPD on placenta (increased ATP and impaired function) [9], and conversely excessive mitochondrial fragmentation can be detrimental [27]. We have now shown that the additional stress of maternal fasting during pregnancy can affect both perinatal parameters and postnatal glucose homeostasis. We predicted that if SIMH were involved in both the response to LPD and to the stress of maternal fasting, the response to stress might be affected (perhaps attenuated) in the offspring of mothers already on LPD. However, no such interaction was apparent (Figures 2 and 3). Such metabolic programming is important because adverse influences that affect fetal growth lead to endocrine and metabolic changes that may benefit the fetus in the short term. However it seems likely that these changes would be maladaptive in adult life, especially in the presence of obesity [28].

Our results, based on mice, are supportive of, but have substantial advantages over, studies of the effects of stress on fetal growth in humans, where stresses are inevitably confounded by other factors [29]. For instance, studies that have found strong causative associations between stress and birth weight have rarely been able to exclude effects of poor nutrition and smoking. Repeated stresses may have effects on both brain structure and postnatal behaviour. In our study we were able to link a single episode of maternal stress at a critical stage of pregnancy both to perinatal parameters and potentially to adult health. Fasting for 16 hours is clearly very stressful for a mouse, much more so than an overnight fast in a pregnant woman. Nevertheless, our findings may be relevant for women who inadvertently undergo significant fasting in human pregnancy.

## MATERIALS AND METHODS

### Animals

Mice were housed in conventional wire-top polycarbonate cages, with a 12:12 light:dark cycle at temperatures between 19-23°C and relative humidity 55±10%. Food and water were offered *ad libitum*. The facility is free of MHV, EDIM, MVM, MPV, PVM, Sendai, TMEV, ectomelia, LCMV, Mad 1 and 2, MCMV, reovirus 3, *Citrobacter rodentium*, *Clostridium piliforme*, *Corynebacterium kutscheri*, *Mycoplasma*, Pasteurellaceae, *Salmonella*, beta-hemolytic streptococci, *Streptococcus pneumoniae*, *Streptobacillus moniliformis*, endoparasites and ectoparasites. *Helicobacter* and MNV are present in this facility. All animals were housed and managed in accordance with the United Kingdom’s Home Office protocols, covered by the Animals (Scientific Procedures) Act 1986. The protocol was approved by the Oxford University Committee on Animal Care and Ethical Review, University of Oxford Medical Sciences division (Project licences 3001526 and 3002208). Seven-week-old female mice from the inbred strain C57BL/6J/OxJR (bred in house but originally obtained from Harlan/Envigo) were acclimatized over a one-week period by handling and recording weights.

In order to assess the effects of nutritional stress and impaired mitochondrial function, the offspring were exposed to a low protein diet (8% as opposed to 20% acid casein based protein corresponding to LPD and NPD) and/or the mitochondrial inhibitor, AZT, *in utero* and for the rest of their postnatal lives (AZT rapidly crosses the human placenta [30]). Following acclimatisation, female mice were maintained on a regime of one of the following: 1) normal (20% protein) diet (NPD); 2) low protein diet - (LPD); 3) NPD and 0.15 mg/ml AZT- (NPDAZT); or 4) LPD and 0.15 mg/ml AZT (LPDAZT), from the age of 8 weeks for 2 weeks prior to mating. Both 8% and 20% protein diets were obtained from Arie Blok, (Netherlands, catalogue numbers 4400.00 and 4400.01 respectively). AZT (Zidovudine, Glaxo Smithkline, UK) was mixed into the drinking water at a concentration of 0.15 mg/ml and this was changed twice weekly. Food and water was given *ad libitum*. Day 0.5 of gestation was determined by detection of a vaginal plug. At embryonic day 18 some animals were sacrificed by cervical dislocation and the concepti collected. In an earlier publication [9] we previously presented data on the stressed NPD and LPD litters (of that publication Figure 1 shows the placental and fetal weights in relation to water content, and Figure 5 the placental mtDNA content).

In work carried out previous to this study, dams were fasted overnight 3 times during pregnancy, at embryonic days 6, 12 and 18. However, only 3/44 (6.8%) litters reached term. This is significantly lower than 41/177 (23%), the rate for pregnancies in which the mothers were not fasted (*p* =0.05). Because of the effect on fecundity, the data therefore presented in this study are from mothers fasted once during pregnancy prior to blood sampling from a tail vein (fasting overnight on E12, henceforth “stressed”) or no fasting (“unstressed”). This is a critical time in pregnancy in the mouse, being soon after the onset of maternal placental vascular perfusion at day 10.5 and coinciding with substantial changes in three marker proteins for reactive oxygen species damage [31]. Baseline food and water consumption was measured just prior to mating by weighing the food and water/AZT in a cage of on average four females, and weighing again 24 hours later. Litters were culled to 4-5 pups by 4 days of age. At 20 weeks, the remaining offspring were fasted overnight prior to sampling under terminal anaesthesia (sodium pentobarbital (Sagatal, Rhone Merieux, Harlow, UK) 60 mg/kg (i.p.)) from the tail vein for a fasting glucose measurement.

### Cell biology methods

BeWo cells [32] and mouse embryonic fibroblasts [27] in which mitochondria were fluorescent (DS-red targeted to mitochondria [32]) were cultured in a 96-well plate and treated for 6 hours in the indicated conditions before fixation with 4% paraformaldehyde (PFA). After DAPI staining, the plate was imaged using the InCell1000 analyser (GE healthcare life sciences, 500 cells per well). Raw images were binarised and mitochondrial morphological characteristics were quantified, notably the degree of branching or mitochondrial form factor (FF) and the mean mitochondria length (in μm). FF is defined as (Pm^2^)/(4πAm), where Pm is the length of mitochondrial outline and Am is the area of mitochondrion [33].

### Statistical analysis

We used SPSS version 22, with the help of the University of Oxford Statistics Department. Analysis of perinatal parameters (placental and fetal weights) was carried out using ANOVA, with dams as the unit of experimentation. In line with previous investigators in this field, analysis of postnatal fasting glucose was carried out on an individual basis.

## SUPPLEMENTARY MATERIALS FIGURES AND TABLE



## Abbreviations

AZT: zidovudine
HPA: hypothalamic-pituitary axis
LPD: low protein diet
MEFs: mouse embryonic fibroblasts
MtDNA: mitochondrial DNA
NPD: normal protein diet
SIMH: stress-induced mitochondrial hyperfusion
