# Supplementary material for: Acute nutritional stress during pregnancy affects placental efficiency, fetal growth and adult glucose homeostasis
Source: Oncotarget. 2017 Nov 25;8(65):109478–86. doi: 10.18632/oncotarget.22695 (PMC5752535; doi:10.18632/oncotarget.22695)
Supplement: Supplementary file 1 [file oncotarget-08-109478-s001.pdf]

Acute nutritional stress during pregnancy affects placental efficiency, fetal growth and adult glucose homeostasis

SUPPLEMENTARY MATERIALS

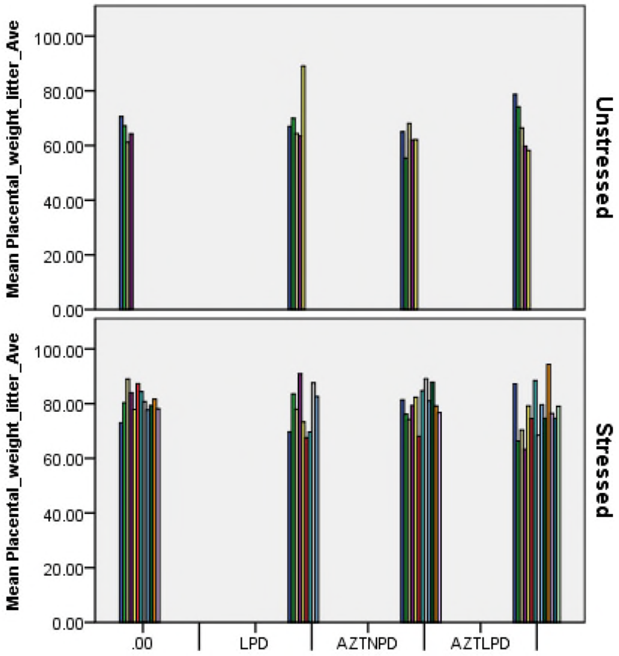

Supplementary Figure 1: Placental weight (mg): each bar represents one litter.

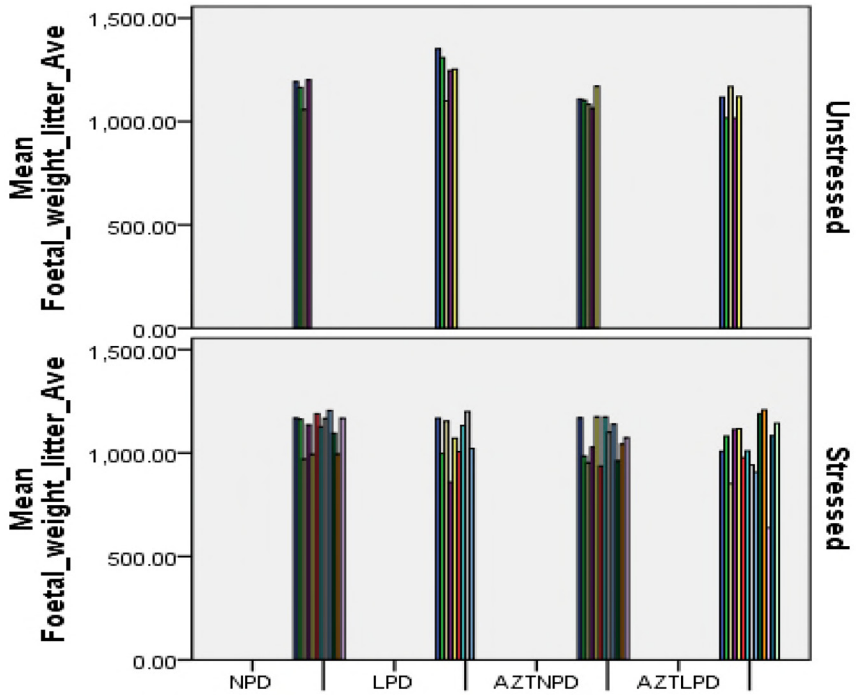

Supplementary Figure 2: Foetal weight (mg): each bar represents one litter.

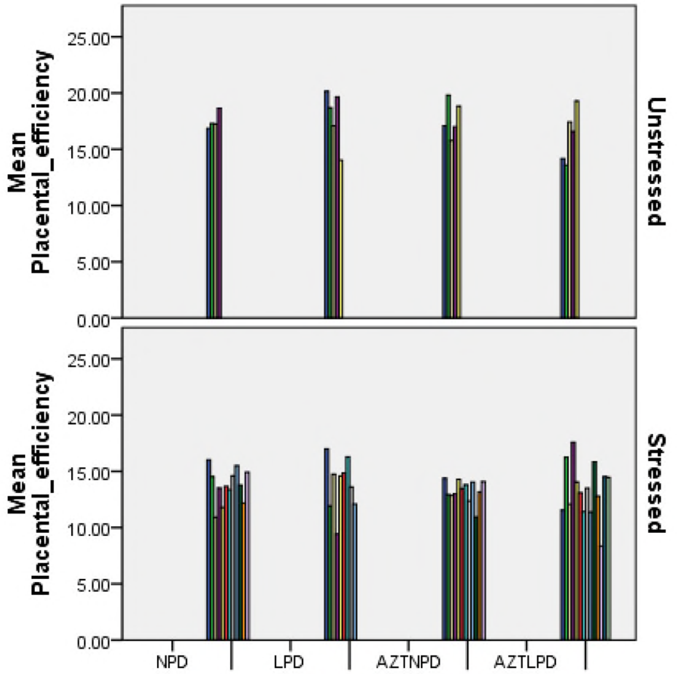

Supplementary Figure 3: Placental Efficiency (foetal weight/embryonic weight): each bar represents one litter.

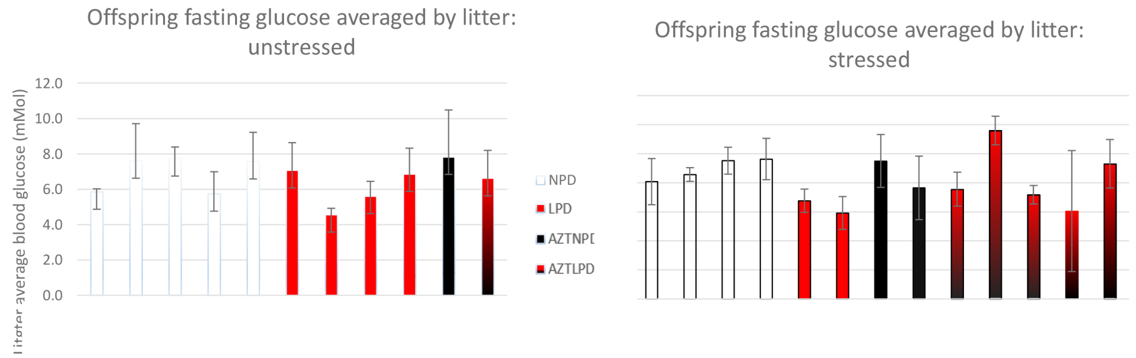

Supplementary Figure 4: Fasting glucose of offspring (mmol) averaged by litter (error bars 1SD). NB difference between stressed and unstressed is not significant using litter as the unit, but is significant when using individuals.

Supplementary Table 1: Data corresponds that that presented in Figure 4

Culture conditions	Be Wo			MEFs		
	Mean uM	N	Std. Deviation	Mean uM	N	Std. Deviation
Glucose (baseline)	3.4	1177	1.22	2.3	1390	0.24
Low Glucose	3.7	1641	1.11	2.5	1988	0.32
Galactose	3.4	1796	0.98	2.3	1372	0.33
Glucose 5% FCS	3.8	1691	1.14	2.5	1493	0.31
Low Glucose 5% FCS	3.7	1367	1.02	2.6	1384	0.33
Glucose - 6mM Glut	3.6	1439	1.12	2.4	2069	0.26
No amino acids	4.0	1297	1.35	2.7	1568	0.53

Mitochondrial length was in all conditions significantly longer in BeWos that MEFs (all $p<0.001$). Mitochondria were the same length at baseline (25mM glucose) and in glucose-free galactose media. In all other conditions, mitochondria were significantly longer than baseline (all $p<0.001$).
